# The next step in Mendelian randomization

**DOI:** 10.7554/eLife.86416

**Published:** 2023-03-09

**Authors:** Matthias Weith, Andreas Beyer

**Affiliations:** 1 Cologne Excellence Cluster on Cellular Stress Responses in Age‐Associated Diseases, and the Institute for Biochemistry, University of Cologne Cologne Germany; 2 Cologne Excellence Cluster on Cellular Stress Responses in Age‐Associated Diseases, the Faculty of Medicine and University Hospital of Cologne, the Center for Molecular Medicine Cologne, and the Institute for Genetics, University of Cologne Cologne Germany

**Keywords:** genetics, metabolomics, gene expression, multiomics, causal inference, Human

## Abstract

Expanding a statistical approach called Mendelian randomization to include multiple variables may help researchers to identify new molecular causes of specific traits.

**Related research article** Auwerx C, Sadler MC, Woh T, Reymond A, Kutalik Z, Porcu E. 2023. Exploiting the mediating role of the metabolome to unravel transcript-to-phenotype associations. *eLife* **12**:e81097. doi: 10.7554/eLife.81097.

Understanding how variations in our genome influence our susceptibility to diseases is one of the most compelling research topics in the life sciences. Researchers have used genome-wide association studies – experiments that analyze the DNA sequences of multiple individuals – to identify statistical relationships between genetic variants and specific human traits, such as susceptibility to a disease or various body parameters.

Despite the success of this approach, major challenges persist. First, associations between variants that are located close to each other within the genome can make it difficult to determine which of these genetic changes are responsible for the phenotype of interest (a problem called linkage disequilibrium). Second, even if specific variants can be identified, it is often not straightforward to determine the molecular mechanism by which they impact the trait (Tam et al., 2019).

To overcome these difficulties, studies often include information about other modalities such as transcriptomes, proteins and metabolites (Emilsson et al., 2008; Fraser and Xie, 2009; Nicolae et al., 2010; Wainberg et al., 2019; Schadt, 2009; Suhre et al., 2011). Some ‘multi-omic’ studies use one modality, or ‘layer’, to confirm changes to another, such as confirming changes in levels of mRNA by measuring the respective protein product. However, there is a shortage of examples of mechanistic links between the different layers (Buccitelli and Selbach, 2020; Wörheide et al., 2021). Now, in eLife, Zoltán Kutalik, Eleonora Porcu and colleagues from the Swiss Institute of Bioinformatics and the University of Lausanne – including Chiara Auwerx as first author – report a new approach that uses a technique called Mendelian randomization to reveal a chain of molecular connections between the transcriptome, metabolome, and high-level physiological traits such as biomarkers associated with kidney health (Auwerx et al., 2023).

Mendelian randomization is considered to be an ‘experiment of nature’, as it uses variations already present in the genetic code to determine if exposure to certain conditions (such as the amount of cholesterol in the blood, or the expression level of a gene) affects a specific trait (for instance, increased susceptibility to heart disease). The genetic variants act as a proxy, or ‘instrument’, for exposures that are difficult or impossible to manipulate in the population being studied. Mediation analysis can then be applied to ask if the exposure is responsible for the effects of the instrumental variable on the trait of interest. However, it is necessary to proceed carefully (Sanderson et al., 2022): for example, the instrumental variable being used should not affect the trait of interest through any other mediator.

The computational framework presented by Auwerx et al. integrates results from genome-wide association studies with data on genetic variants that affect the level of transcripts or the composition of metabolites. These variants are typically referred to as eQTL (short for expression quantitative trait loci) and mQTL (metabolite QTL), and can be derived from separate population cohorts, allowing researchers to tap into the vast resources of information that are already available.

First, causal links between transcripts and metabolites are established using overlapping mQTL and eQTL as instrumental variables. Causal effects of metabolites on traits of interest are then determined in the same manner using mQTL and genetic variants identified in genome-wide association studies. The next step in the framework is purely based on this established causality: transcripts that causally affect trait-modifying metabolites have to be causally linked to the same trait, resulting in transcript-metabolite-trait triplets (Figure 1). A statistical calculation, known as multivariate Mendelian randomization, is then performed on these triplets using the metabolite-associated variants as the instrumental variable. This determines what proportion of change in the outcome is a result of the transcript directly (or via unknown mediators) impacting the trait, and what proportion is the result of changes in the level of the metabolite mediating the relationship between them.

**Figure 1.**
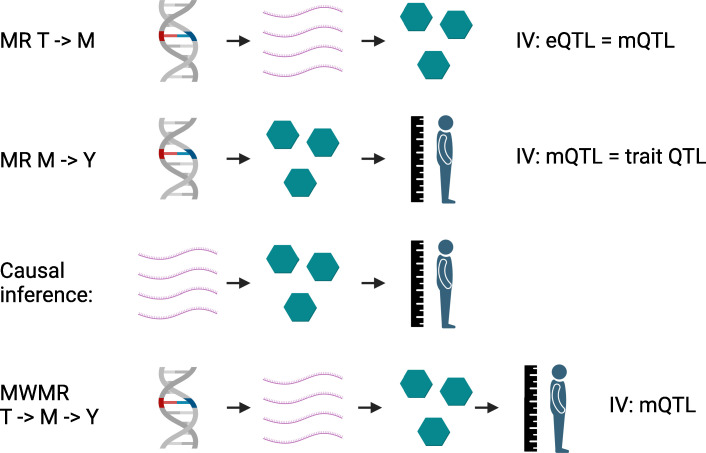
Mendelian randomization with multiple variables. In the first step, Mendelian randomization calculations establish causal links between: (i) transcripts (T; pink chains) and metabolites (M; green hexagons) using eQTL and mQTL as instrumental variables (IV; first row); (ii) metabolites and various phenotypes (Y, such as height), using mQTL and the genetic variants associated with the traits as instrumental variables (second row). These causal links are then overlapped to establish causal triplets (third row). These causal triplets are subsequently analyzed in another Mendelian randomization-based calculation, which evaluates the effect of the respective mQTL on the levels of the transcripts, metabolites and traits of the triplet (fourth row). From this multivariate Mendelian randomization (MWMR), the proportion of transcript changes that directly effect a trait, and the proportion that cause an effect via metabolites, can be inferred. eQTL: expression quantitative trait loci; mQTL: metabolite quantitative trait loci.

Image credit: Figure created using BioRender (CC BY 4.0).

Auwerx et al. highlight an intriguing example of genetic variants affecting the transcription of a citrate-exporting protein encoded by a gene called *ANKH* that has been implicated in mineralization disorders. The resulting change to the export of citrate seems to affect the level of calcium present in the serum of individuals – a connection that was not detected when only transcript levels were correlated with the calcium trait.

By extending the Mendelian randomization approach to include two modalities (transcripts and metabolites), this new framework can detect causal relationships that could not be identified by comparing the genome wide association data to a single modality only. It also provides new insights into how the transcript impacts the phenotype through metabolic changes. With multi-omics studies increasing further in size, it is highly probable that even more advanced statistical approaches may become feasible in the future.
